# Development of an Anti-Zika and Anti-Dengue IgM ELISA Assay: Evaluation of Cross Reactivity and Validation

**DOI:** 10.3390/tropicalmed7110348

**Published:** 2022-11-03

**Authors:** Helena Cerutti, Giulia Tesi, Claudia Soldatini, Tommaso Bandini, Marinunzia Castria, Alessandra Brogi

**Affiliations:** 1 R&D Biotechnology Research, DIESSE Diagnostica Senese S.p.A. Società Benefit, Via Fiorentina, 1-ssc/o Toscana Life Sciences, 53100 Siena, Italy; 2 R&D Development, DIESSE Diagnostica Senese S.p.A. Società Benefit, Via delle Rose, 10, 53035 Monteriggioni, Italy

**Keywords:** capture Elisa, IgM, Zika, dengue, cross-reaction

## Abstract

Zika and dengue viruses (ZIKV and DENV) have been considered major global threats to humans in the past decade. The two infections display similar epidemiological and clinical manifestations. They are transmitted by the same primary vector, accounting for the co-circulation of the two viruses in regions where they are endemic. Highly specific and sensitive serological assays that are able to detect ZIKV and DENV antibodies (Abs) during the acute and convalescent phases of infections would help to improve clinical management and disease control. We report the development and characterisation of two monoclonal Abs, the ZIKV 8-8-11 and the DENV 8G2-12-21, which recognise the Zika non-structural protein 1 (NS1) and the dengue virus type 2 envelope protein, respectively. Both mAbs were used to set up enzyme-linked immunosorbent assays (ELISAs) specific for the detection of anti-Zika immunoglobulin M (IgM) and anti-dengue IgM and whose performance was similar to commercially available kits. These kits, intended to be used with the CHORUS Instruments, are rapid and require ≤50 µL of human serum. These tests could represent an affordable and reliable option for the rapid diagnosis of both ZIKV and DENV infections in developing countries, where these flaviviruses are endemic.

## 1. Introduction

Zika virus (ZIKV) and dengue virus (DENV) belong to the *Flaviviridae* family and both have been reported as major global threats to humans over the past decade [1]. The two infections display similar epidemiological and clinical manifestations, but their pathogenesis differ [2].

ZIKV is a single-stranded ribonucleic acid (RNA) virus which is primarily transmitted through the bite of the mosquito of the genus *Aedes* [3]. However, the transmission of ZIKV can also occur through blood transfusion, sexual contact, during pregnancy and peri-partum [3,4,5]. While most ZIKV acute infections are asymptomatic, the most frequently reported symptoms are rash, fever, arthralgia, non-purulent conjunctivitis, myalgia, and headache. The later consequences of ZIKV infection are more serious. Indeed, different studies report a strong association (odds ratio > 34) between the development of Guillain-Barré syndrome [6] and previous ZIKV infection(s). More importantly, ZIKV infection during pregnancy can cause different foetal abnormalities, including microcephaly and ocular alterations [7,8,9]. Epidemiological studies from French Polynesia outbreaks and from Brazil, suggest that the greatest risk of microcephaly is when the infection occurs during the first trimester of pregnancy [5,10,11,12,13].

The diagnosis of ZIKV infection relies on the detection of the virus by reverse-transcriptase polymerase chain reaction (RT-PCR) and the detection of immunoglobulin (Ig)M antibodies (Abs) in serum that become detectable by the end of the first week after symptom onset, which is then followed by the development of neutralising IgG Abs [14]. The United States (U.S.) Centers for Disease Control and Prevention (CDC) recommends IgM testing in symptomatic pregnant women, on serum that is collected from 2–12 weeks after clinical manifestations and within 14 days of symptom onset if RT-PCR is negative [15]. Similar recommendations are in place for asymptomatic pregnant women; samples should be tested using RT-PCR and IgM anti-ZIKV can be detected within 14 days or from 2–20 weeks after potential exposure, respectively [16].

Dengue represents the most important disease-causing arbovirus in tropical and subtropical regions, with a 30-fold increase in cases over the past 5 decades [17]. DENV is a single-stranded positive-sense RNA, which is mainly transmitted by *Aedes aegypti*. The increase in transmission is likely to be because of urbanisation and globalisation [18]. Infection can be caused by 4 different virus serotypes (DENV1–4). While 75% of patients are generally asymptomatic, the clinical manifestation of symptomatic patients can range from mild to severe, from a flu-like syndrome (dengue fever with fever, nausea, vomiting, rash, aches, and pains) to dengue shock syndrome, with severe bleeding [19]. The World Health Organization classifies dengue into dengue (with/without warning signs) and severe dengue. The sub-classification has been carried out to promote close clinical observation and to reduce the risk to develop severe dengue [20]. During early infection (<5 days), the diagnosis of dengue infection can be performed by detection of viral RNA by nucleic acid amplification tests or the detection of non-structural protein 1 (NS1) antigens. Following this period (>5 days after infection), diagnosis relies on the detection of Abs [21]. IgM first increases then tends to wane overtime, while IgG generally increases during primary infections and convalescence. IgG levels will remain stable, so subjects became immunised to homologous but no other virus serotypes [22]. During secondary and tertiary infections with a different serotype, non-neutralising IgG levels increase with viremia and the enhanced IgG levels seem to favour viral replication [23].

ZIKV and DENV are transmitted by the same primary vector, which is likely to account for the co-circulation of the two viruses in the many regions where they are found to be endemic. Moreover, ZIKV and DENV have been reported to share ~45–55% protein homology and during natural infections, Abs are induced both against envelope protein (EP) and against the viral proteins NS1, NS3, and NS5 [24]. Interestingly and more intriguingly, a different antibody binding patterns to structural and non-structural proteins after acute ZIKV infection in different body fluids has also been reported recently [25]. The EP is a flavivirus structural glycoprotein that facilitates receptor binding on the host cell membrane. Three principal, functional domains, EDI, EDII and EDIII, are exposed. EPs also display icosahedral arrangement meaning that 90 envelope dimers coat the viral surface and switch conformation in relation to virus maturation. DENV and ZIKV EP share ~55% similarity in amino acid sequences. Conversely, the glycosylation of EPs appears to differ, with a single glycosylation site for ZIKV EP (Asn 154), and two glycosylation sites (Asn67 and Asn153) for DENV EP, respectively. Therefore, the similarity between DENV and ZIKV EPs allow these glycoproteins to act as the major surface antigen, and cross-reactive Abs are frequently detected by the enzyme-linked immunosorbent assay (ELISA) at the same rate [26]. 

Considering the non-specific clinical manifestations of acute ZIKV and DENV infections, their diagnosis relies on molecular and serological testing. However, the differential diagnosis can be a challenge given the documented immunological cross-reactivity between the ZIKV and DENV, resulting in misinterpretation of the results due to the cross-reactive nature of Abs elicited by both infections [27,28]. 

Rapid, highly specific, and sensitive serological assays can detect ZIKV and DENV Abs during the acute and convalescent phases of infections, which would improve clinical management and disease control. In this manuscript, we describe the characterization of monoclonal Abs (mAbs) specific to the NS1 protein of ZIKV and the EP of DENV, which are reported to be the most important antigens able to trigger an immunological response against ZIKV and DENV, respectively.

## 2. Materials and Methods

Recombinant Zika NS1 protein (cat# R01636); DENV EP (aa 281-675) (cat# R01557); DENV type 1 NS1 (cat# R01656), DENV type 2 NS1 (cat# R01657), DENV type 3 NS1(cat# R01658), DENV type 4 NS1 (cat# R01663) tagged with histidine, and DENV type 2 antigen (cat# R02220) were purchased from Meridian Bioscience, Inc. (Memphis, TN, USA).

Immunisation and hybridoma generation. Anti-Zika Ab. Five 6- to 8-week-old BALB/c mice were immunised with 20 µg ZIKV NS1 recombinant protein emulsified in complete Freund’s adjuvant and administered intra-peritoneally (i.p.). Booster injections were given after 2 weeks (20 µg, i.p.), after 5 weeks (10 µg, i.p.), and after 6 weeks [15 µg, intravenously, extracellular vesicles (e.v.)]. Based on the humoral response from a blood sample taken before the last immunisation dose, the highest BALBc/responder [according to an indirect-ELISA (iELISA)] was selected as a donor of splenocytes to produce hybridomas. Splenocytes were obtained 3 days after the final booster. Hybridomas were obtained by fusion with Sp2/0-Ag14 myeloma cells (ClonaCell™-HY Hybridoma Kit, STEMCELL Technology, Vancouver, Canada) following vendor instruction. The iELISA screening of over 500 positively secreting hybridomas, led to the selection of the ZIKV 8-8-11 clone.

Anti-dengue Ab. Five 6- to 8-week-old BALB/c mice were immunised with 20 µg DENV EP emulsified in complete Freund’s adjuvant, administered i.p. Booster injections were given after 2 weeks (20 µg, i.p.), after 5 weeks (10 µg, i.p.), after 6 weeks (10 µg, i.p.), and after 7 weeks (15 µg, e.v.). Based on the humoral response from a blood sample taken before the last immunisation dose, the highest BALBc/responder (according to iELISA) was selected as a donor of splenocytes for hybridoma production. Hybridomas were obtained by fusion with Sp2/0-Ag14 myeloma cells (ClonaCell™-HY Hybridoma Kit; STEMCELL Technology), following vendor instruction. The screening of over 500 positively secreting hybridomas, carried out by iELISA led to the selection of the clone DENV 8G2-12-21.

mAbs purification. Anti-ZIKV and -DENV Abs were purified by protein G affinity chromatography (“HiTrap Protein G HP Ab purification columns”, Cytiva, Dassel, Germany) from hybridoma culture supernatants, grown in a disposable bioreactor for Ab and recombinant protein production (Corning^®^ CELLine Disposable Bioreactor for Ab and recombinant protein production, Corning, New York, NY, USA).

Isotyping of immunoglobulin. The isotype of mAbs was determined using the Mouse mAb Isotyping Reagents (Merck KGaA, Darmstadt, Germany) according to the manufacturer’s instructions.

Human sera. Human sera, both positive and negative controls, used in this study are commercially available and were obtained by AbBaltis (Sittingbourne, Kent, ME9 8LR, UK), Biomex (BIOMEX GmbH, Heidelberg, Germany), Diaserve (Diaserve Laboratories GmbH, Iffeldorf, Germany), Biochemed (Winchester, VA, USA), Cerba (Cerba HealthCare, St Ouen L’Aumone, Paris, France), Abo Phar-maceutical (Abo Pharmaceutical, San Diego, CA, USA), ZeptoMetrix (Buf-falo, NY, USA) PNCQ (Rio de Janeiro, Brazil). Positive or negative sera characterized with commercial CE marked kits for the “presence” or “absence” of IgM antibodies against dengue or Zika virus, were purchased. All samples were frozen and kept at −20 °C until the time of simultaneous comparison on the test and reference method.

IBL International Zika virus IgM micro-capture ELISA (IBL International, Hamburg, Germany) is a qualitative immune-enzymatic method of detecting specific IgM-Class Abs against ZIKV based on the ELISA µ-Capture technique. The kit consists of microplates coated with anti-human IgM-class Abs and a labelled Zika virus antigen. Patients’ sera were tested following manufacturer’s instruction. The results, expressed as arbitrary units, were interpreted as followed: <9 as negative; 9–11 as equivocal, and >11 as positive.

Panbio Dengue IgM Capture ELISA (Abbott, CA, USA). This is an ELISA performed in 96 well plates which are pre-coated with anti-human IgM, followed by the addition of the recombinant dengue 1–4 antigens. All of the samples were processed following the manufacturer’s instructions. The absorbance average for the calibrator tested was multiplied by the calibrator factor to get the cut-off value. The results were calculated in index and interpreted as followed: <0.9 as negative; 0.9–1.1 as equivocal and, >1.1 as positive.

Euroimmun Anti-Dengue virus ELISA (IgM) (Euroimmun, PerkinElmer Company, Lübeck, Germany). This ELISA test kit provides an indirect semi-quantitative in vitro assay for human Abs of the IgM class against dengue viruses. The test kit contains microtiter strips coated with dengue virus antigens. Then, the specific serum IgM will be detected using labelled anti-human IgM Abs. Results were evaluated by calculating a ratio of the extinction value of the control or patient sample over the extinction value of the calibrator and are interpreted as follows: ratio < 0.8 as negative, between ≥0.8 to <1.1 as borderline; ratio ≥ 1.1 as positive.

Statistical analysis. All of the analyses were performed by the Analyze-it (Microsoft Excel). Sensitivity, specificity, negative predict values (NPV), and positive predictive value (PPV) with 95% confidence intervals (CI) of the CHORUS kits were calculated against the “reference” kits results. Agreement between tests results were assessed by calculating Cohen’s Kappa coefficient (k), interpreted as follows: values ≤0 indicate no agreement, 0.01–0.20 as none to slight, 0.21–0.40 as fair, 0.41–0.60 as moderate, 0.61–0.80 as substantial, and 0.81–1.00 as almost perfect agreement.

Ethical statement. Animal were housed, handled, and treated at Emozoo srl (Casole d’Elsa, SI, Italy). All procedures involving mice were conducted in compliance with institutional animal care guidelines and followed national and international directives (D.L. 4 March 2014, No. 26; directive 2010/63/EU of the European parliament and council; Guide for the Care and Use of Laboratory Animals, United States National Research Council, 2011). Experimental protocols were carried out under the rules stated at Emozoo, project # 843/2015-PR. 

## 3. Results

The antigen selection, both for ZIKV and DENV viruses, relied on the different type of antigens whose sequences were available, on their homologies among the different Zika and dengue virus strains, and on epidemiological data on virus prevalence [29,30]. In addition, for each parameter, a preliminary screening was performed testing some positive sera with different antigens for ZIKV and DENV (including the four different serotypes). Most of the reactivity was against the two selected antigens NS1 for ZIKV and native dengue virus type 2 for DENV; therefore they were selected for the immunocomplexes used in the diagnostic kits described (data not shown). 

***Production and characterisation of anti-ZIKV NS1 and anti-DENV EP mAbs.***

With the aim to obtain hybridomas that secrete specific Abs against the ZIKV NS1 and DENV EP proteins, mice were immunised following the protocols described in the material and methods section. Hybridomas were generated by the fusion of spleen cells from immunised mice and myeloma cells. Screening of the hybridoma clones were performed by indirect iELISA with the same antigens used for immunisation.

The hybridoma clones ZIKV 8-8-11 and DENV 8G2-12-21 were selected due to highly expressing high affinity mAbs against ZIKV NS1 and DENV EP. The isotypes of both mAbs are shown in Table 1 and in both cases consist of IgG1, as indicated by the higher OD value. 

***Specificity of the mAbs***

To evaluate the specificity of the developed mAbs, we analysed the reactivity of the mAb ZIKV 8-8-11 towards the NS1 proteins of the 4 dengue serotypes and towards the histidine tag of the recombinant immunisation protein. The control was carried out by iELISA method, using plates coated with the antigens which were to be evaluated and the mAbs were used as the detector. The mAb did not cross react with the pool of NS1 or with histidine (Table 2). Similar considerations can be taken for the anti-DENV 8G2-12-21, that seems to be specific for the DENV EP and does not cross react with the ZIKV EP or histidine tag (Table 2).

These results indicate that ZIKV 8-8-11 and DENV 8G2-12-21 mAbs are highly specific for NS1-Zika and EP-dengue, respectively, and do not cross-react with dengue NS1 and Zika EP, respectively.

***Development of an enzyme immunoassay method for the determination of anti-Zika and anti-dengue virus IgM Abs in human serum with a single-use device processed by CHORUS TRIO instrument.***

Using the ZIKV 8-8-11 and DENV 8G2-12-21 mAbs, we developed two immunoassays for the qualitative determination of anti-Zika and anti-dengue virus IgM Abs in human serum. The CHORUS Zika IgM Capture and CHORUS Dengue IgM Capture tests were set up to be used with the DIESSE Diagnostica Senese CHORUS system, a fully automated instrument capable of simultaneously processing 30 samples. The results of the CHORUS Zika IgM and Dengue IgM Capture are obtained in 140 min [31]; the user only adds 50 µL of serum in a mono test device containing all the reagents that are necessary to carry out the test. The test relies on the MAC-ELISA (IgM Ab capture ELISA) principle on which anti-human IgM mAbs (MAB 5M, generated against the polypeptide subunit corresponding to the heavy chain) are bound to the solid phase; IgM bind to anti-IgM mAbs following incubation with a diluted sample. Briefly, after washing to remove the excess proteins, incubation is carried out with the antigen bound to specific mAbs conjugated with horseradish peroxidase (Zika NS1 antigen with ZIKV 8-8-11 for Zika virus and dengue antigen type 2 with DENV 8G2-12-21 for dengue virus). The serum Ab concentration is proportional to colour and the results are expressed as index value, where the test is deemed positive with a result >1.1; negative when the result is <0.9, and uncertain when the result is between 0.9–1.1.

The specificity of the assay was tested using commercially available sera that were positive for ZIKV, DENV, and other viruses. Table 3 shows the distribution of the samples used for the cross-reactivity studies on CHORUS Zika IgM and Dengue IgM Capture devices using positive sera to ZIKV, DENV and West Nile virus. Supplementary Tables S1 and S2 report the individual data obtained. As shown, cross-reactions were detected with only few samples positive to West Nile Virus (a virus that also belongs to *Flaviviridae*). Simultaneous or recent infections with another flavivirus cannot be excluded.

***Comparison with other commercially available assays***

A total of 159 human serum samples were analysed by both the CHORUS Zika IgM capture assay and Zika virus IgM micro-capture ELISA (IBL international). The comparison between the two tests is shown in Table 4; the degree of agreement between the two methods was excellent as demonstrated by the k value (Cohen’s coefficient) of 0.88.

An excellent agreement (k value 0.82) was also obtained when comparing the CHORUS Dengue IgM capture assay with the Panbio Dengue IgM Capture ELISA, a capture method that uses the 4 dengue antigens recombinant protein, and which is considered the gold standard (Table 5). Moreover, when the Euroimmun anti-Dengue virus ELISA (IgM) was used for the analysis of discordant samples (no 13: 9 false positives and 4 false negatives), a further increased in diagnostic sensitivity and specificity was found with a k value of 1 because all presumed false results have been confirmed as real positives and negatives (Table 6).

## 4. Discussion

ZIKV and DENV infections have been reported as major global threats to humans over the past decade [1]. ZIKV infections have received particular attention in 2015 after the outbreak in Brazil [32] and the cumulating evidence of the association between the ZIKV infection and severe sequelae, such as the Guillain-Barre syndrome in adults [6] and the malformations occurring in foetuses from infected pregnant women [8,9]. The severe constellation of foetal and birth defects is known as congenital Zika syndrome, which mainly affects the central nervous system [8] prompted the World Health Organization to declare the ZIKV infection a Public Health Emergency of International Concern in 2016 [33].

Infections by other flaviviruses, i.e., DENV, have a similar clinical manifestation to infections caused by ZIKV and considering that often both infections can occur in coincident outbreaks, the differential diagnosis is of paramount importance, especially in pregnant women. Serological tests for the identification of specific anti-ZIKV-IgM and anti-DENV-IgM are available, but the reported cross-reactive interference of the different flaviviruses accounts for possible false positive results. To the best of our knowledge, there are no approved commercially available clinical diagnostic tests which correctly identify ZIKV without cross-reactivity against other flaviviruses infections [34]. The development of a specific diagnostic assay for ZIKV and DENV is an urgent need, especially in epidemic settings where two or more flaviviruses are present.

Most diagnostic kits available for the detection of anti-IgM against ZIKV and DENV are ELISA-based tests, whose overall performance relies on the specificity of the immune complex (antigen and its related Ab) used to detect specific IgM present in patient sera [35]. In this study, we have developed and characterised two mAbs, ZIKV 8-8-11 and DENV 8G2-12-21, which recognise the Zika NS1 protein and the dengue virus type 2 EP, respectively. Both mAbs were used to set up an automated ELISA test applied to the CHORUS TRIO instrument by DIESSE Diagnostica Senese. The CHORUS Zika and Dengue IgM Capture tests, set up with these labelled mAbs in the immune complex, were demonstrated to be highly specific for the detection of anti-Zika IgM and anti-DENV IgM, respectively. In fact, the CHORUS Zika IgM Capture was tested in sera samples from patients who were infected with ZIKV, and all 15 positive cases were detected, and no cross-reactivity was found from the 35 sera samples that were positive for anti-DENV IgM used in the testing process. Similar results were obtained with the CHORUS Dengue IgM Capture that could recognise all 52 sera samples that were positive for DENV and no cross-reactivity was observed in anti-ZIKV positive sera. These data are important considering that ZIKV and DEV viruses share high degree of structural and sequence homology and co-circulate in many regions of the world and could help in a differential diagnosis [36]. Furthermore, only a small cross-reactivity against West Nile, another virus of *Flaviviridae* family was observed, although simultaneous or recent infections with another flavivirus cannot be excluded. More importantly were the findings that both tests were comparable with the commercially available kits. In fact, the degree of agreement was excellent. The described kits are devices to be used in the CHORUS Instruments and allow for rapid quantification (less than 2.5 h), using no more than 50 µL of human serum sample.

## 5. Conclusions

In conclusion, we have developed and characterised two specific mAbs against ZIKV NSI and DENV EP, that were used for the setup of two automated ELISA tests. 

These kits complete a panel of tests for the diagnosis of vector-borne tropical infections. In particular, kits for the diagnosis of West Nile Virus have been developed at the same time as the dengue and Zika tests.

Even more data are necessary to use these tests to differentiate active infections from convalescent samples, and they could represent reliable options for rapidly diagnosing both ZIKV and DENV infections, particularly in developing countries, where these flaviviruses are endemic. The samples can be analysed simultaneously for both parameters in a safe and simple manner for laboratory operators. The possibility to perform a diagnosis that can differentiate between these two infections, whose clinical manifestations are similar, and/or in asymptomatic patients, in areas where the two viruses are endemic, will greatly help in their epidemiological surveillance and could be a valid tool to prevent the severe sequelae of Zika infections in pregnant women.

## Figures and Tables

**Table 1:** Isotypes of ZIKV 8-8-11 and DENV 8G2-12-21 mAbs.

mAb	IgG1	IgG2a	IgG2b	IgG3	IgM
**ZIKV 8-8-11**	1.049	0.069	0.072	0.086	0.067
**DENV 8G2-12-21**	1.317	0.121	0.117	0.105	0.047

DENV: dengue virus; IgG: immunoglobulin G; IgM: immunoglobulin M; mAb: monoclonal antibody; ZIKV: Zika virus. Results are expressed as OD (Optical Density) measured at 450 nm wavelength.

**Table 2:** Specificity of the ZIKV 8-8-11 and DENV 8G2-12-21 mAbs.

**ZIKV 8-8-11**	**ZIKV NS1**	**Pool DENV NS1**	**His**
	2.117	0.054	0.085
**DENV 8G2-12-21**	**DENV EP**	**ZIKV EP**	**His**
	2.117	0.196	0.172

DENV: dengue virus; NS1: non-structural protein 1; EP: envelope protein; His: histidine; ZIKV: Zika virus. All the reported values are absorbance values (optical density, OD, value at 450 nm wavelength).

**Table 3:** Specificity of the CHORUS Zika and Dengue IgM Capture assays.

	**Results**	**ZIKV** **(N Total Samples = 159)**	**DENV** **(N Positive Samples = 35)**	**WNV** **(N Positive Samples = 10)**
**CHORUS Zika IgM Capture**	Positive	15	0	2
	Equivocal	2	0	0
	Negative	142	35	8
	**Results**	**DENV** **(N Total Samples = 187)**	**ZIKV** **(N Positive Samples = 15)**	**WNV** **(N Positive Samples = 10)**
**CHORUS Dengue IgM Capture**	Positive	52	0	3
	Negative	135	15	7

DENV: dengue virus; IgM: immunoglobulin M; WNV: West Nile virus; ZIKV: Zika virus.

**Table 4:** Comparison between the CHORUS and IBL IgM capture assays.

		Zika Virus IgM Micro-Capture ELISA (IBL International)			% of Sensitivity (95% CI)	% of Specificity (95% CI)	k Value (95% CI)
		+	−	Total	93.5% (70.0–98.7)	98.6% (95.1–99.6)	0.88 (0.83–0.98)
**CHORUS Zika IgM Capture**	+	14	2	16			
	−	1	142	143			
	Total	15	144	159			

CI: confidence interval; ELISA: enzyme-linked immunosorbent assay; IgM: immunoglobulin M; ZIKV: Zika virus.

**Table 5:** Comparison between the CHORUS and Panbio Dengue IgM Capture ELISA.

		Panbio Dengue IgM Capture ELISA			% of Sensitivity (95% CI)	% of Specificity (95% CI)	k Value (95% CI)
**CHORUS Dengue IgM Capture**		+	−	Total	91.5 (80–96.6)	96.3 (88.1–96.5)	0.82
	+	43	9	52			
	−	4	130	134			
	Total	47	139	186			

CI: confidence interval; ELISA: enzyme-linked immunosorbent assay; IgM: immunoglobulin M.

**Table 6:** Analysis of discordant samples with Euroimmun anti-Dengue Elisa (IgM).

		Euroimmun Anti-Dengue Virus ELISA IgM		
**CHORUS Dengue IgM Capture**		+	−	Total
	+	9	0	9
	−	0	4	4
	Total	9	4	13

## Data Availability

The data presented in this study are available on request from the corresponding author.

## Supplementary Materials

Supporting information can be downloaded at: https://www.mdpi.com/article/10.3390/tropicalmed7110348/s1, Table S1. Specificity of the CHORUS Zika IgM capture; Table S2. Specificity of the CHORUS Dengue IgM capture kit.
