# Supplementary material for: Development of an Anti-Zika and Anti-Dengue IgM ELISA Assay: Evaluation of Cross Reactivity and Validation
Source: Trop Med Infect Dis. 2022 Nov 3;7(11):348. doi: 10.3390/tropicalmed7110348 (PMC9693240; doi:10.3390/tropicalmed7110348)
Supplement: Supplementary file 1 [file tropicalmed-07-00348-s001.zip › tropicalmed-1932909-supplementary.pdf]

Tropical Medicine and Infectious Disease

MDPI

Supplementary Materials: Development of an Anti-Zika and Anti-Dengue IgM ELISA Assay: Evaluation of Cross Reactivity and Validation

Table S1. Specificity of the CHORUS Zika IgM capture.

Sample Code	Factor	Index	Result	Expected Result
t248	West Nile	0.5	N	<0.9
t249	West Nile	0.6	N	<0.9
t250	West Nile	0.5	N	<0.9
t1445	West Nile	0.2	N	<0.9
t1446	West Nile	0.3	N	<0.9
t1448	West Nile	0.2	N	<0.9
t1449	West Nile	0.2	N	<0.9
t846	West Nile	0.7	N	<0.9
ZK9	West Nile	3,9	**P**	<0.9
ZK10	West Nile	5,5	**P**	<0.9
1	Dengue	0.4	N	<0.9
2	Dengue	0.7	N	<0.9
3	Dengue	0.8	N	<0.9
4	Dengue	0.5	N	<0.9
5	Dengue	0.7	N	<0.9
6	Dengue	0.8	N	<0.9
7	Dengue	0.4	N	<0.9
8	Dengue	0.6	N	<0.9
9	Dengue	0.9	N	<0.9
10	Dengue	0.5	N	<0.9
11	Dengue	0.6	N	<0.9
12	Dengue	0.6	N	<0.9
13	Dengue	0.4	N	<0.9
14	Dengue	0.4	N	<0.9
15	Dengue	0.3	N	<0.9
16	Dengue	0.3	N	<0.9
17	Dengue	0.3	N	<0.9
18	Dengue	0.4	N	<0.9
19	Dengue	0.4	N	<0.9
20	Dengue	0.4	N	<0.9
21	Dengue	0.4	N	<0.9
22	Dengue	0.3	N	<0.9
23	Dengue	0.4	N	<0.9
24	Dengue	0.4	N	<0.9
25	Dengue	0.4	N	<0.9
26	Dengue	0.4	N	<0.9
27	Dengue	0.5	N	<0.9
28	Dengue	0.4	N	<0.9
29	Dengue	0.3	N	<0.9
30	Dengue	0.3	N	<0.9
31	Dengue	0.4	N	<0.9

Sample Code	Factor	Index	Result	Expected Result
32	Dengue	0.4	N	<0.9
33	Dengue	0.5	N	<0.9
34	Dengue	0.4	N	<0.9
35	Dengue	0.4	N	<0.9

N: negative; P: positive.

Table S2. Specificity of the CHORUS Dengue IgM capture kit.

Sample Code	Factor	Index	Result	Expected Result
WNG1	West Nile IgM Positive	0.2	N	<0.9
WNG3	West Nile IgM Positive	0.2	N	<0.9
WNG4	West Nile IgM Positive	0.2	N	<0.9
TRINA1446	West Nile IgM Positive	2.1	**P**	<0.9
TRINA1448	West Nile IgM Positive	1.7	**P**	<0.9
TRINA1449	West Nile IgM Positive	0.3	N	<0.9
TRINA 248	West Nile IgM Positive	0.7	N	<0.9
TRINA 249	West Nile IgM Positive	0.7	N	<0.9
TRINA 250	West Nile IgM Positive	0.7	N	<0.9
Trina 1445	West Nile IgM Positive	1.2	**P**	<0.9
Z02	Zika IgM Positive	0.3	N	<0.9
ZK 5	Zika IgM Positive	0.3	N	<0.9
ZK 6	Zika IgM Positive	0.2	N	<0.9
ZK 7	Zika IgM Positive	0.3	N	<0.9
ZK 8	Zika IgM Positive	0.3	N	<0.9
ZK 9	Zika IgM Positive	0.6	N	<0.9
ZK 10	Zika IgM Positive	0.8	N	<0.9
ZK51	Zika IgM Positive	0.3	N	<0.9
ZK61	Zika IgM Positive	0.2	N	<0.9
ZK71	Zika IgM Positive	0.3	N	<0.9
ZK81	Zika IgM Positive	0.2	N	<0.9
ZK52	Zika IgM Positive	0.2	N	<0.9
ZK62	Zika IgM Positive	0.2	N	<0.9
ZK72	Zika IgM Positive	0.2	N	<0.9
ZK82	Zika IgM Positive	0.2	N	<0.9
VZM ST2	Varicella IgM Positive	0.2	N	<0.9
VZM ST3	Varicella IgM Positive	0.3	N	<0.9
VZM 394	Varicella IgM Positive	0.2	N	<0.9
RVM ST2	Rubella IgM Positive	0.3	N	<0.9
RVM ST3	Rubella IgM Positive	0.3	N	<0.9
RVM 380	Rubella IgM Positive	0.2	N	<0.9
RVM 381	Rubella IgM Positive	0.3	N	<0.9
CMV M 220	Cytomegalovirus IgM Positive	0.2	N	<0.9
CMV M 221	Cytomegalovirus IgM Positive	0.3	N	<0.9
CMV M 224	Cytomegalovirus IgM Positive	0.3	N	<0.9
CMV M 792	Cytomegalovirus IgM Positive	0.2	N	<0.9
CMV M 793	Cytomegalovirus IgM Positive	0.3	N	<0.9
CMV M 794	Cytomegalovirus IgM Positive	0.3	N	<0.9
CMV M 808	Cytomegalovirus IgM Positive	0.2	N	<0.9
CMV M 815	Cytomegalovirus IgM Positive	0.3	N	<0.9
CMV M 823	Cytomegalovirus IgM Positive	0.2	N	<0.9

IgM: immunoglobulin; N: negative; P: positive
